# Lung Cancer Mortality and Topography: A Xuanwei Case Study

**DOI:** 10.3390/ijerph13050473

**Published:** 2016-05-06

**Authors:** Hongyan Ren, Wei Cao, Gongbo Chen, Junxing Yang, Liqun Liu, Xia Wan, Gonghuan Yang

**Affiliations:** 1 State Key Laboratory of Resources and Environmental Information System, Institute of Geographic Sciences and Natural Resources Research, Chinese Academy of Sciences, Beijing 100101, China; caowei@igsnrr.ac.cn (W.C.); yangajx@126.com (J.Y.); 2 Institute of Basic Medical Sciences, Chinese Academy of Medical Sciences, 5, Dong Dan San Tiao, Beijing 100005, China; chengongbo2008@126.com (G.C.); liuliqun726@163.com (L.L.); wanxiasnake@163.com (X.W.)

**Keywords:** Xuanwei, lung cancer mortality, spatiotemporal variation, topography

## Abstract

The epidemic of lung cancer in Xuanwei City, China, remains serious despite the reduction of the risk of indoor air pollution through citywide stove improvement. The main objective of this study was to characterize the influences of topography on the spatiotemporal variations of lung cancer mortality in Xuanwei during 1990–2013. Using the spatially empirical Bayes method, the smoothed mortality rate of lung cancer was obtained according to the mortality data and population data collected from the retrospective survey (1990–2005) and online registration data (2011–2013). Spatial variations of the village-level mortality rate and topographic factors, including the relief degree of land surface (RDLS) and dwelling conditions (VDC), were characterized through spatial autocorrelation and hotspot analysis. The relationship between topographic factors and the epidemic of lung cancer was explored using correlation analysis and geographically weighted regression (GWR). There is a pocket-like area (PLA) in Xuanwei, covering the clustered villages with lower RDLS and higher VDC. Although the villages with higher mortality rate (>80 per 10^5^) geographically expanded from the center to the northeast of Xuanwei during 1990–2013, the village-level mortality rate was spatially clustered, which yielded a persistent hotspot area in the upward part of the PLA. In particular, the epidemic of lung cancer was closely correlated with both RDLS and VDC at the village scale, and its spatial heterogeneity could be greatly explained by the village-level VDC in the GWR model. Spatiotemporally featured lung cancer mortality in Xuanwei was potentially influenced by topographic conditions at the village scale.

## 1. Introduction

Xuanwei City (formerly Xuanwei County, Xuanwei) of Yunan Province in southwest China is widely known for having the highest lung cancer mortality rate among all Chinese counties. This has mainly been attributed to indoor air pollution caused by polycyclic aromatic hydrocarbons (PAHs), particulate matter, and silica emitted from “smoky coal” burnt for household heating or cooking without adequate ventilation [1,2,3]. In the 1970s, the government encouraged local residents to improve their stoves, and this stove improvement project was completed in the late 1980s. Household stoves have been improved in Xuanwei in the past 30 years and indoor exposure to benzopyrene and particulate matter in observed houses decreased [4,5]. There are no obvious differences between type of stove and coal among the various communities in Xuanwei. However, the mortality rate of lung cancer in different communities is still obviously different [6,7,8], which suggested that the other factors may affect the spatial pattern of lung cancer mortality and morbidity in Xuanwei.

Since the 1970s, the epidemiological, clinical, and etiopathological features of lung cancer in Xuanwei have been studied extensively [1,2,9,10,11,12,13], increasing our understanding of the distribution of this disease and its relation to environmental factors. However, spatiotemporal patterns and the potential influences of particular geo-environmental conditions on the mortality rate of lung cancer in Xuanwei remain poorly understood at a fine spatial scale. In this context, the aim of this study was to characterize the spatiotemporal distribution of the mortality rate of lung cancer at the village scale and then to explore its relationship with geo-environmental factors in Xuanwei. These approaches may allow us to better describe and understand the association between the epidemic of lung cancer and environmental conditions.

## 2. Methods

### 2.1. Study Area

**Figure 1:**
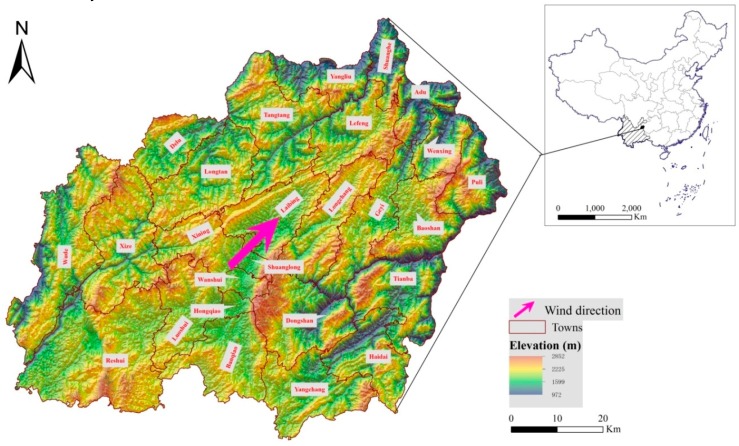
Illustration of the study area and basic topographic information.

Xuanwei is a mountainous region covering 6052.96 km^2^ in northeastern Yunan Province, with prevailing southwesterly winds (Figure 1). The region is the home of plentiful coal, iron, copper, and other mining operations, especially smoky coal. During 1990–2013, the town-level administrative divisions were changed from Rongcheng, Jingwai, and Chengguan Town to Xi’ning, Shuanglong, Wanshui, and Hongqiao Town in the urban area. However, the village-level divisions remained the same as before. This spatial scale, therefore, is comparable between different years and was applied for this study.

### 2.2. Data Collection

#### 2.2.1. Topographic Conditions

**Figure 2:**
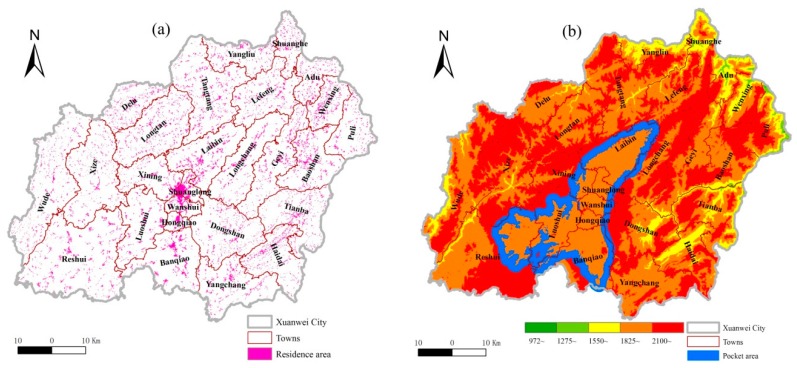
Residential area (**a**) and featured topography (**b**) in Xuanwei.

Residential area data for 2011–2013 were collected from local land departments in Xuanwei (Figure 2a). The village-level residential area (VRA) was then obtained for all villages using the Zonal Statistics tool in the software ArcGIS 10.0 (ESRI, Redlands, CA, USA). Topography was also characterized in terms of categorized height (Figure 2b) derived from digital elevation model data with a spatial resolution of 100 × 100 m (available on www.resdc.cn). The relatively flat areas (RFA) were defined based on the range of height (equal to or less than 30 m) in the surrounding 3 × 3 grids (about 0.09 km^2^), and extracted for each village by the focal range tool in ArcGIS 10.0. Subsequently, the residential areas located in the RFAs were extracted by means of overlay analysis tool in ArcGIS 10.0 and then assigned as VRA_f_ for each village. To indicating the dwelling condition (VDC) that means the topographic suitability of residence in each village, the ratio of VRA_f_ in the residential area was accordingly calculated for each village as follows:

(1) $$\text{VDC}={\text{VRA}}_{f}/\text{VRA}$$

In addition, topography in Xuanwei was further characterized at the village scale in terms of the relief degree of land surface (RDLS) that represents the general condition of the average altitude, the proportions of plain land surface, and the range of elevation in a specified area. In this study, RDLS was calculated using the following formula:

(2) RDLS=ALT/1000+Range(H)×[1−P(A)/A]/BM

where ALT is the mean altitude (m) in each village, Range(H) is the difference between the maximum and minimum altitude in each village, A is the village-level area (km^2^), P(A) is the total area (km^2^) of relatively flat regions in each village, and BM denotes the height (500 m) of baseline mountains throughout the mainland of China [14]. The process of determining RDLS has been described in detail by Feng, *et al.* [14].

#### 2.2.2. Lung Cancer Mortality Data

The mortality and population data used in this paper were reported by Chen, *et al.* [8]. In brief, mortality data during 1990–1992 and 2004–2005 were obtained from the 2nd and 3rd National Retrospective Sampling Survey on Mortality. Mortality data for 2011–2013 were directly extracted from the online reports of death registration system (DRS) in the Center of Disease Control and Prevention in Xuanwei. Particularly, considering the existence of under-reported deaths in the local DRS, a survey on underreported deaths was conducted and mortality data during 2011–2013 were adjusted according to regional rates of underreporting [15]. To reduce the spatial instability of the village-level mortality rate of lung cancer caused by the size of village-level population (below 30,000), the Spatial Empirical Bayes technique (based on a locally varying reference mean) was applied to smooth the mortality rate of lung cancer for each village using the software of OpenGeoDA1.2.0 (available on https://geodacenter.asu.edu/software/downloads). The villages were then divided into five groups over each period according to the mortality rate relative to the age standardized mortality rate of lung cancer in rural areas of China (1 to 4-fold) as described by Chen, *et al.* [8].

#### 2.2.3. Statistical Analysis

Pearson’s correlation was used in the analysis of the village-level mortality rate of lung cancer associated with VDC and RDLS, to improve understanding of the relationship between this epidemic and topographic factors on the village scale in Xuanwei. In this study, *p* values of less than 0.01 were considered to indicate statistical significance. All analysis was conducted using SAS 9.0 software (SAS Institute Inc., Cary, NC, USA).

### 2.3. Spatial Analysis

#### 2.3.1. Spatial Autocorrelation

Spatial autocorrelation analysis is frequently utilized to explore the spatial patterns of incidence or mortality in terms of Moran’s *I* because of its excellent statistical power [16,17,18]. According to the following formula [19], Moran’s *I* is produced by standardizing the spatial autocovariance by the variance of the data using a measure of the connectivity of the data:

(3) $$I=\frac{N\sum_{i}\sum_{j}\omega_{ij}\left(x_{i}-\bar{x}\right)\left(x_{j}-\bar{x}\right)}{\left(\sum_{i}\sum_{j}\omega_{ij}\right)\sum_{i}{\left(x_{i}-\bar{x}\right)}^{2}}$$

where *N* is the total number of units (villages), *ω_ij_* is an element of the weight matrix (N×N), *ω_ij_* is a weight which can be defined as follows: when the village *i* is contiguous to village *j*, the weight *ω_ij_* is given the weight of 1; otherwise the weight *ω_ij_* is given the weight of 0. *x*_i_ and *x*_j_ are given attributes of *i*-village and *j*-village respectively, and $\bar{x}$ is the mean of the given attributes (*i.e.*, smoothed mortality rate, VDC, or RDLS). Moran’s *I* value ranges from −1 to 1. Generally, a higher positive Moran’s *I* value represents a tendency toward clustering, suggesting that adjacent villages have similar levels. In contrast, a lower negative value indicates a tendency toward dispersal which suggests that the villages with a high level are located next to villages with a low level. A fuller description is provided by Anselin and Getis [19].

#### 2.3.2. Hotspot Analysis

Compared with the capability of the spatial autocorrelation in indicating the spatial patterns (global clustering or dispersing) of the villages, hotspot analysis can further capture the detailed spatial patterns (local clustering) by means of locating the “hot” (higher values) or “cold” (lower values) clusters [20,21]. In this study, Gettis-OrdG*i* * [22] was chosen to identify the locations of statistically significant hot spots and cold spots based on the following formula:

(4) $$G_{i}^{*}=\frac{\sum_{j=1}^{n}\omega_{i,j}x_{j}-\bar{X}\sum_{j=1}^{n}\omega_{i,j}}{S\sqrt{\frac{\left[n\sum_{j=1}^{n}\omega_{i,j}^{2}-{\left(\sum_{j=1}^{n}\omega_{i,j}\right)}^{2}\right]}{n-1}}}$$

where *x*_j_ is the attribute value for *j*-village, *ω_ij_* is the spatial weight between village *i* and *j*, *n* is equal to the total number of villages and:

(5) $$\bar{X}=\frac{\sum_{j=1}^{n}x_{j}}{n}$$

(6) $$S=\sqrt{\frac{\sum_{j=1}^{n}x_{j}^{2}}{n}-{\left(\bar{X}\right)}^{2}}$$

The Gi* statistic is a *z*-score so no further calculations are required. A high *z* score and small *p* value for a village indicates a significant hot spot. A low negative *z* score and small *p* value indicates a significant cold spot. The higher (or lower) the *z* score, the more intense the clustering. A *z* score near zero implies no local clustering.

Geographically weighted regression (GWR) is an extension of the traditional multiple linear regression toward a local regression in which the regression coefficients are specific to a location rather than global estimates [23,24,25]. In this study, GWR was also used to integrate the topographic factors (VDC and RDLS) to explain the spatiotemporal variations of lung cancer mortality at the village scale. The process of GWR modeling in this study is described in detail in the supplementary files. The analysis of spatial autocorrelation, hotspots, and GWR were performed using the ArcGIS 10.0 software.

## 3. Results

### 3.1. Descriptive Statistics

Xuanwei is a typically mountainous region with an elevation from 972 to 2852 m (Figure 1) with few RFAs (1315.28 km^2^, or about 22%) and few residential areas (164.88 km^2^, about 3%). The overlapping residential areas (79.98 km^2^), as assessed by RFA, accounted for 49% of all residential areas in Xuanwei, suggesting that about one half of the residential areas were located in the uneven area. At the village scale, local villages had unfavorable dwelling conditions due to the high mean value of RDLS and relatively low mean value of VDC (Table 1). In addition, about 50% of all the land in Xuanwei is within regions at the 4th (approximately 1825 m) altitude level (Figure 2b). In particular, there is a clear pocket-like area (PLA) with an average altitude of 2023 m is surrounded by a ring-like belt (buffer zone width of 2 km) with higher average altitude (2192 m, Figure 2b).

In 1990–2013, the village-level mortality rate of lung cancer in Xuanwei showed clear temporal variations. The mean value of village-level mortality rate dramatically increased from 38.97 per 10^5^ (1990–1992) to 88.18 per 10^5^ (2004–2005) and 98.75 per 10^5^ (2011–2013), as shown in Table 1. Moreover, the differences in village-level mortality rate of lung cancer increased between 1990 and 2005 and decreased between 2005 and 2013 (Table 1).

### 3.2. Topographic Characteristics

**Figure 3:**
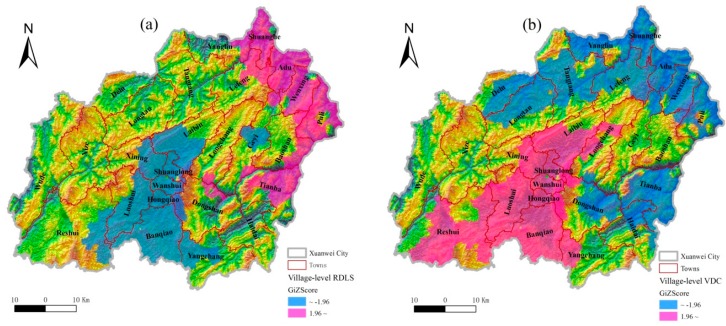
Spatial clustering of the village-level RDLS (**a**) and VDC (**b**). RDLS and VDC denote the relief degree of land surface and the dwelling condition, respectively.

At the village scale, Xuanwei is clearly characterized by the spatial distribution of RDLS and VDC. In terms of Moran’s *I* (Table 2), both RDLS and VDC are spatially clustered at the village scale. Most of the villages in the central-southern part had low RDLS (Figure 3a) and high VDC (Figure 3b). These villages happened to be distributed in PLA. This showed that PLA is a special region with not only cold spots of RDLS but also hotspots of VDC at the village scale.

### 3.3. Spatiotemporal Variations of Lung Cancer Mortality

**Figure 4:**
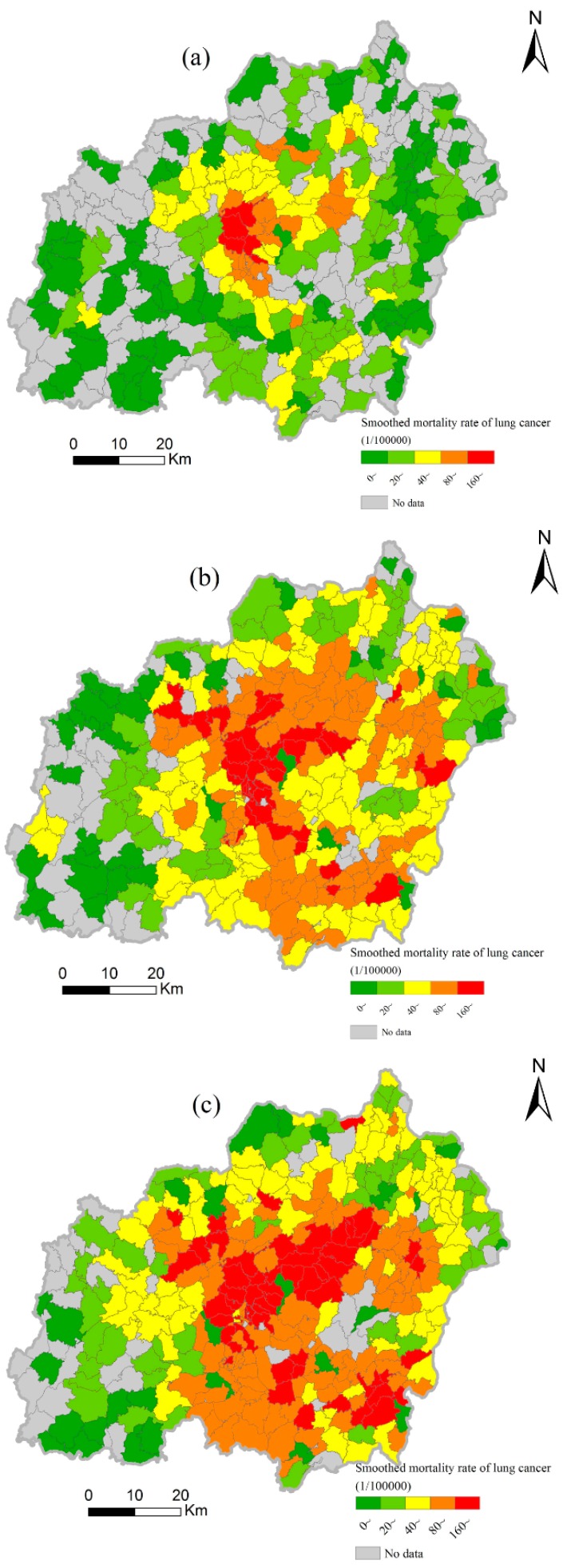
Spatial disparities of the village-level mortality rate of lung cancer during 1990–1992 (**a**); 2004–2005 (**b**); and 2011–2013 (**c**).

**Figure 5:**
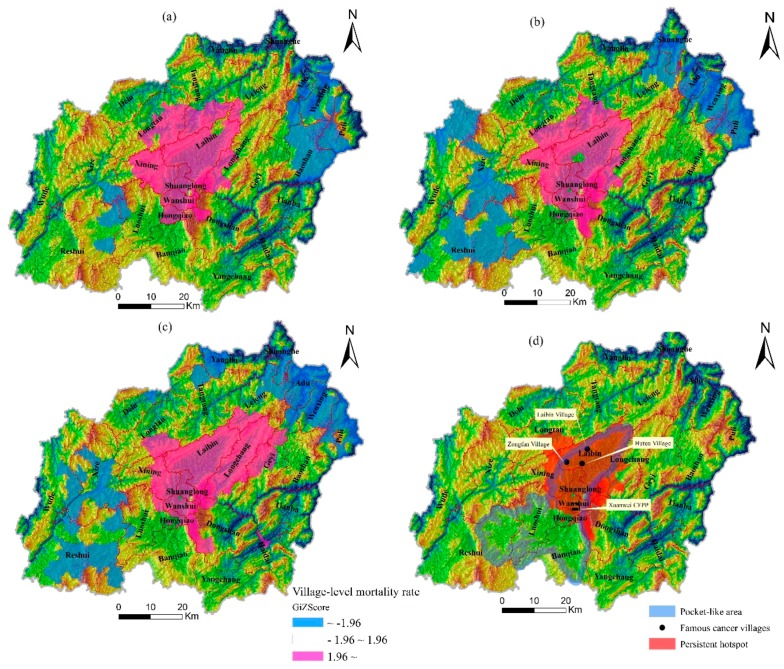
Hotspot regions of the village-level mortality rate of lung cancer 1990–1992 (**a**); 2004–2005 (**b**); 2011–2013 (**c**); and 1990–2013 (**d**). CFPP is the abbreviation for coal-fired power plant.

The mortality rate of lung cancer in Xuanwei presented clear temporal and spatial variations at the village scale. In 1990–1992, only 26 villages in the central region of Xuanwei had a relatively high mortality rate of lung cancer (>80 per 10^5^, Figure 4a). The number of villages with relatively high mortality rate (>80 per 10^5^) increased during 2004–2005 (125) and 2011–2013 (151), yielding a clear expansion from the center toward the northeast and southeast (Figure 4b,c). Moreover, the village-level mortality rate was spatially clustered, according to the spatial autocorrelation coefficients (Table 2). Hotspots of the village-level mortality rate in 1990–2013 were uniformly distributed in the central part of Xuanwei, although they displayed a gradual contraction in the northwest and a clear expansion in the northeast direction (Figure 5a–c). Accordingly, there was a constant region (dark pink polygons in Figure 5d) with higher risk of lung cancer due to its larger *z* values during 1990–2013.

### 3.4. Relationship Between Lung Cancer and Topography

In the case of spatial distribution, the constant region with higher *z* values of the village level mortality rate were largely overlapped by the PLA-related villages with higher (VDC) and lower (RDLS) *z* values. In addition to the similar spatial distribution, the mortality rate of lung cancer was also closely correlated with topographic factors at the village scale. Table 3 shows that the village-level mortality rate was negatively correlated with RDLS at the significance level of 0.01 and the correlations became increasingly stronger between 1990 and 2013. In contrast, VDC results suggested positive impacts (r = 0.33, *p* < 0.01) on this epidemic. In addition, RDLS was significantly negatively correlated with VDC at the village level (r = −0.71, *p* < 0.01). These results show that the mortality rate of lung cancer in Xuanwei was spatially and temporally related to the special topographic conditions over recent decades.

Furthermore, the topographic factors employed in GWR models accounted for about 40% or more of spatial variations in lung cancer mortality in Xuanwei, although *R*^2^ and adjusted *R*^2^ values of GWR models decreased during 1990–2013 (Table S1). Considering the interpretability of GWR models, the combination of RDLS and VDC failed to perform better in comparison to use of a single factor (RDLS or VDC). Because the lowest AICs and highest adjusted *R*^2^ (or *R*^2^) values were present in the third stage (2011–2013), the model including only the village-level VDC was most capable of explaining the spatial variations of mortality rate of lung cancer. According to the values of the standardized residual (Std Resid) from 2011–2013 (Figure S1), the villages with Std Resid values in the range of −2 to 2 account for 94.7% of the whole city, which indicates that the relationship between the village-level mortality rate and VDC is stable. Overall, the spatial variations of lung cancer mortality in Xuanwei during 1990–2013 were appropriately interpreted by the village-level topographic factors included in the GWR models.

## 4. Discussion

This study characterized the spatial and temporal relationships between the mortality rate of lung cancer and topographic factors at the village scale in Xuanwei during 1990–2013. To the best of our knowledge, this study is the first quantitative and qualitative investigation on the association between this epidemic and topographic factors, which can supply useful references for environmentalists and epidemiologists who focus on the epidemic of lung cancer in this city.

Xuanwei is a typically mountainous region with few RFAs, widely distributed narrow valleys, and continuous mountains. There is a special region (PLA) with spatially clustered villages with lower RDLS and higher VDC. In some special areas with environmental pollution, the plain zones enclosed by the relatively higher terrain often possess better capability to accumulate contaminants [26]. Because of the plentiful coal deposits, coal burning is ubiquitous in Xuanwei for cooking, household heating, and industrial production [1,2,3]. Accordingly, we suggest that the PLA in Xuanwei acts as a “container” of air pollution, especially under the adverse meteorological conditions caused by the prevailing southwesterly winds (Figure 1). Therefore, local people in the PLA are subjected to the risk of air pollution mainly caused by coal burning.

Studies have shown that the risk of local people from lung cancer has been somewhat reduced after a citywide project of improving stoves with installed chimneys in 1970–1980 [4,10,27,28]. However, patterns of death due to lung cancer have not changed and some communities continue to suffer from the high lung cancer mortality, especially in the central area of Xuanwei [6,8]. Similarly, our analysis showed that the upper part of PLA in Xuanwei was a persistent hotspot region of village-level mortality rate (Figure 5d). In this region, there are three prominent villages which are lung cancer hot-spots: Hutou, Laibin, and Zongfan [6,11,13,29]. These results imply that the upper part of PLA is the most important zone because of the high mortality rate of lung cancer and requires more effective interventions for preventing and controlling this epidemic in the future.

Previous studies have stated that the death rates from some diseases (e.g., lung cancer, cardiovascular) are usually correlated with some topographic factors, such as numerical elevation or altitude [30,31,32,33,34]. The current study also demonstrated that spatial variations of lung cancer mortality in Xuanwei were quantitatively associated with topographic characteristics at the village scale. In addition, the VDC-GWR model was able to interpret the spatial heterogeneity of lung cancer mortality. In Xuanwei, the PLA enclosed by surrounding mountains could easily act to restrict dispersion of the contaminants, including total suspending particles (TSP), PAHs, nitrogen oxides (NO_x_), sulfur dioxide (SO_2_), vented from chimneys, especially under adverse weather conditions. In addition, people are willing to reside in RFAs even though they are often surrounded by mountains. Therefore, the larger the village-level VDC in Xuanwei, the higher the mortality rate of lung cancer the village possessed. With the exception of air pollution caused by coal burning, these adverse conditions (mountainous land and prevailing southwesterly winds) are not environmental factors that can be eliminated. Hence, we suggest that the replacement of coal by clean energy should be made as soon as possible.

As mentioned above, both larger mortality rate of lung cancer and higher VDC were simultaneously observed in the upper part of PLA. Coincidentally, the Xuanwei coal-fired power plant has been present for about 60 years near the split line between the persistent hotspot and PLA (Figure 5d). Recently, this heat-engine plant has been increasingly considered as an important source of air pollution in this city [35,36]. Together with its particular location and the prevailing southwesterly winds, increasing production capacity (from 50 to 1200 MW in the past decades) can explain the importance of this source. Accordingly, we cautiously speculate that the spatial expansion of hotspot areas of lung cancer mortality in Xuanwei in 1990–2013 was probably always associated with this plant. Nevertheless, we suggest that the concentration and spatial distribution of released ingredients by this plant should be further investigated in detail, and that much cleaner production of this power plant should be continuously accelerated.

A few limitations of this study warrant mention. First, the spatiotemporal relationship between topographic conditions and carcinogenic components (e.g., PAHs) in the ambient air should be further explored because air pollution is the main reason for lung cancer mortality in Xuanwei, although current results preliminarily satisfied the association between lung cancer death rate and topography. Second, the spatial unit of both lung cancer mortality and topographic factors in this study requires an even finer resolution than the village scale for geographically representing them and modeling their relationship. Finally, many more environmental factors (e.g., the pollution emission volume of each research unit) and socioeconomic conditions (e.g., the domestic income level at an appropriate scale) should be further integrated into GWR models. This should improve the capability of risk factors to explain the spatial variations of lung cancer mortality in this city, especially in PLA, which would yield more effective environmental health interventions for local authorities.

## 5. Conclusions

In summary, the spatiotemporal characteristics of lung cancer mortality in Xuanwei are potentially influenced by topographic conditions at the village scale. We suggest that more effective interventions for preventing and controlling this epidemic should be concentrated on the upper area of the PLA. Furthermore, the replacement of coal by cleaner energies for local people and reforming clean production by the Xuanwei coal-fired power plant should be made as soon as possible in this city. This study may be useful for continuing investigations of lung cancer epidemiology and its relation to environmental factors in this region. In addition, these approaches may be valuable to local hygiene authorities for use in the assessment of the influences of outdoor air pollution on the epidemic of lung cancer.

## Figures and Tables

**Table 1:** Descriptive statistics of the village-level mortality rate and topographic factors in 1990–2013.

Descriptive Statistics	Smoothed Mortality Rate of Lung Cancer (Per 105)			RDLS			VDC
	1990–1992	2004–2005	2011–2013	1990–1992	2004–2005	2011–2013	2011–2013
Mean	38.97	88.18	98.75	2.87	2.89	2.88	0.40
Range	264.45	470.27	458.32	2.41	2.51	2.56	1.00
Standard Deviation	40.57	78.10	86.10	0.46	0.51	0.51	0.34

Note: The number of villages with both reported mortality and population data was 225, 307, and 318 during 1990–1992, 2004–2005, and 2011–2013. RDLS means the relief degree of land surface; VDC indicates the village-level dwelling condition.

**Table 2:** Spatial autocorrelation of the village-level mortality rate and dwelling condition.

Parameters of Spatial Features	Smoothed Mortality Rate of Lung Cancer (Per 105)			RDLS	VDC
	1990–1992	2004–2005	2011–2013	2011–2013	2011–2013
Moran’s I	0.71	0.62	0.49	0.90	0.91
Z Score	31.46	27.02	20.44	37.15	44.72
*p*-value	0.00	0.00	0.00	0.00	0.00

Note: The number of villages with both reported mortality and population data was 225, 307, and 318 during 1990–1992, 2004–2005, and 2011–2013. RDLS means the relief degree of land surface; VDC indicates the village-level dwelling condition.

**Table 3:** Correlation between the village-level mortality rate and topographic factors in 1990-2013.

Variables	1990–1992	2004–2005	2011–2013
RDLS	−0.34 **^‡^**	−0.36 **^‡^**	−0.37 **^‡^**
VDC	/	/	0.33 **^‡^**

Note: The symbols of **^‡^** mean the significance level of 0.01. RDLS means the relief degree of land surface; VDC indicates the village-level dwelling condition.

## Supplementary Materials

The following are available online at www.mdpi.com/1660-4601/13/5/473/s1, Figure S1: Spatial distribution of standardized residuals from the VDC-GWR model for the prediction of the village-level SMR in Xuanwei during 2011–2013., Table S1: Parameters of GWR models for the prediction of the village-level SMR in 1990-2013.
