# Supplementary material for: Lung Cancer Mortality and Topography: A Xuanwei Case Study
Source: Int J Environ Res Public Health. 2016 May 6;13(5):473. doi: 10.3390/ijerph13050473 (PMC4881098; doi:10.3390/ijerph13050473)
Supplement: Supplementary file 1 [file ijerph-13-00473-s001.pdf]

SupplementaryMaterials: Lung Cancer Mortality and Topography: A Xuanwei Case Study

Hongyan Ren, Wei Cao, Gongbo Chen, Junxing Yang, Liqun Liu, Xia Wan and Gonghuan Yang

Methods

Geographically Weighted Regression (GWR) Modeling

Given the spatiotemporal heterogeneity of lung cancer mortality, the related factors may affect this epidemic in different ways and to different degrees. This can be analyzed using a GWR model. This model is an extension of the traditional multiple linear regression toward a local one in which the regression coefficients are specific to a location rather than global estimates [1,2]. Moreover, this model is based on the spatial non-stationarity, which is common in spatial process: an explanatory variable might be highly relevant in one application but seemingly irrelevant in another. In contrast, parameters describing the same relationship might be negative in some application but positive in others, and the same model might replicate data accurately in one system but not in another [3]. The GWR model embeds the data's spatial location into the regression parameter. The local estimation of the parameters with GWR is expressed using the following equation [4]:

$$y_i = \beta_0(u_i, v_i) + \sum_{k=1}^{n} \beta_{ik}(u_i, v_i)x_{ik} + \varepsilon_i \qquad (i=1,\ 2,\ \ldots,\ m) \tag{s1}$$

where (u_i, v_i) denotes the spatial location of village *i* in Xuanwei, $y_i$is the dependent variable (SMR of lung cancer) at location *i*, x_{ik} represents independent variables including VDC and RDLS of village *i*, β_0 is the intercept, β_{ik} is the correlation coefficient for x_{ik}, which is to be estimated, and $\varepsilon_i$is the random error. Therefore every village in this study has a set of specific parameters to reflect the relationship between the village-level SMR of lung cancer and its explanatory variables. Together with the R^2 and adjusted R^2 values, the Akaike Information Criterion (AIC) was selected through an iterative optimization process as an indicator for evaluting the capability of independent variables (in the models) in explaining the spatial variations of lung cancer mortality [5].

Table S1. Parameters of GWR modes for the prediction of the village-level SMR in 1990–2013.

Periods	RDLS			RDLS+VDC			VDC		
	R^2	adj-R^2	AICs	R^2	adj-R^2	AICs	R^2	adj-R^2	AICs
1990–1992	0.54	0.47	2179.87	/	/	/	/	/	/
2004–2005	0.49	0.42	3403.14	/	/	/	/	/	/
2011–2013	0.44	0.40	3587.63	0.42	0.37	3603.29	0.51	0.42	3587.34

Note: GWR represents the geographically weighted regression; adj-R^2 is the adjusted R square. SMR represents smoothed mortality rate of lung cancer; RDLS means the relief degree of land surface; VDC indicates the village-level dwelling condition.

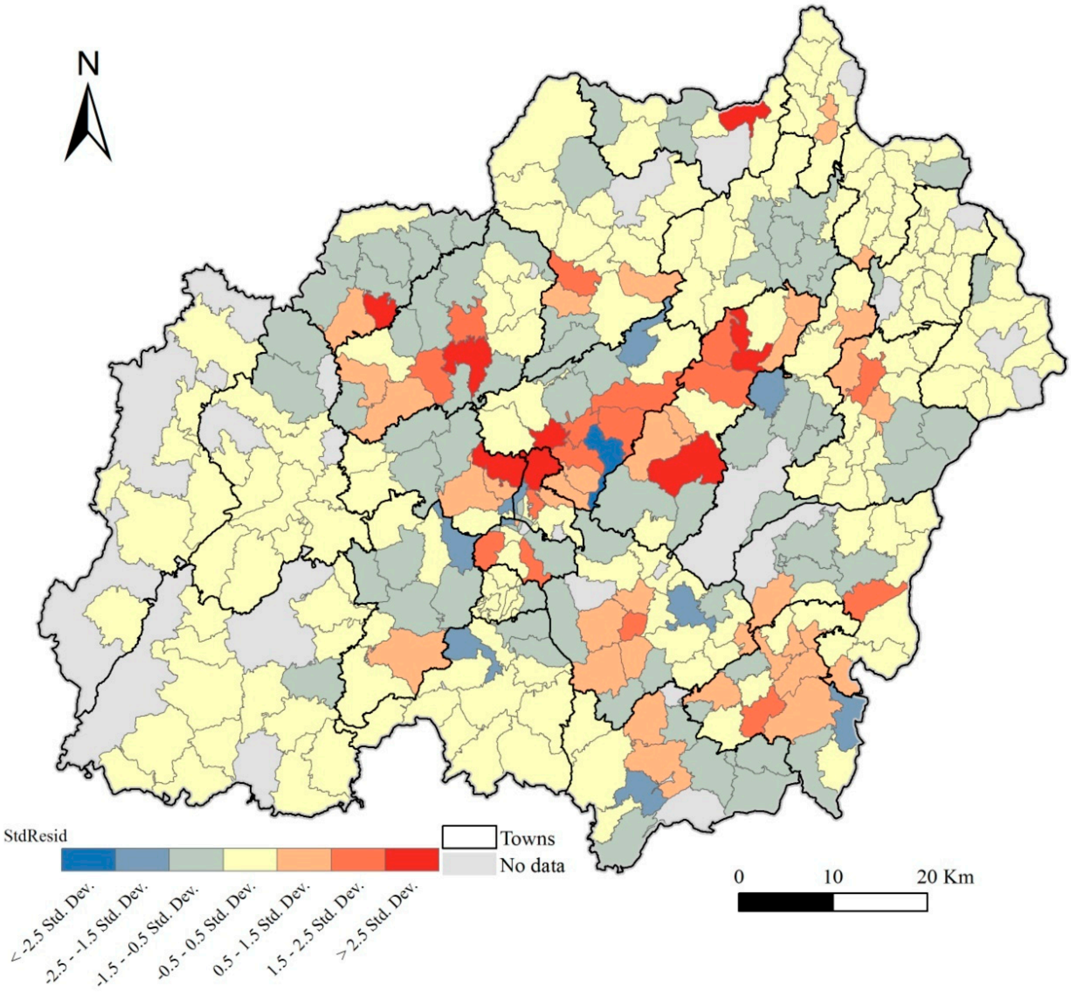

Figure S1. Spatial distribution of standardized residuals from the VDC-GWR model for the prediction of the village-level SMR in Xuanwei during 2011–2013. GWR and VDC represent geographically weighted regression and the village-level dwelling condition.

References

1. Anselin, L.; Syabri, I.; Kho, Y. Geoda : An introduction to spatial data analysis. *Geograph. Anal.* **2005**, *38*, 5–22.
2. Unwin, D.; Unwin, A. Local indicators of spatial association—Foreword. *J. R Stat. Soc.* **1998**, *47*, 413–413.
3. Fotheringham, A.S.; Brunsdon, C.F.; Charlton, M.E. Geographically weighted regression: The analysis of spatially varying relationships. *Am. J. Agric. Econ.* **2004**, *86*, 554–556.
4. Yang, L.X.; Yang, G.S.; Yao, S.M.; Yuan, S.F. A study on the spatial heterogeneity of grain yield per hectare and driving factors based on esda-gwr. *Economic Geogr.* **2012**, *32*, 120–126.
5. Li, S.; Ren, H.; Hu, W.; Lu, L.; Xu, X.; Zhuang, D.; Liu, Q. Spatiotemporal heterogeneity analysis of hemorrhagic fever with renal syndrome in china using geographically weighted regression models. *Int. J. Environ. Res. Public Health* **2014**, *11*, 12129–12147.

© 2016 by the authors; licensee MDPI, Basel, Switzerland. This article is an open access article distributed under the terms and conditions of the Creative Commons by Attribution (CC-BY) license (http://creativecommons.org/licenses/by/4.0/).
